# Antihyperlipidemic Effects of *Sesamum indicum* L. in Rabbits Fed a High-Fat Diet

**DOI:** 10.1155/2013/365892

**Published:** 2013-09-04

**Authors:** Sedigheh Asgary, Mahmoud Rafieian-Kopaei, Somayeh Najafi, Esfandiar Heidarian, Amirhossein Sahebkar

**Affiliations:** ^1^Isfahan Cardiovascular Research Center, Isfahan Cardiovascular Research Institute, Isfahan University of Medical Sciences, Isfahan, Iran; ^2^Medical Plants Research Center, Shahrekord University of Medical Sciences, Shahrekord, Iran; ^3^Physiology Research Center, Isfahan Cardiovascular Research Center, Isfahan Cardiovascular Research Institute, Isfahan University of Medical Sciences, Isfahan, Iran; ^4^Clinical Biochemistry Research Center, Shahrekord University of Medical Sciences, Shahrekord, Iran; ^5^Biotechnology Research Center, Mashhad University of Medical Sciences, Mashhad, Iran

## Abstract

The present study aimed to investigate the anti-hyperlipidemic effects of sesame in a high-fat fed rabbit model. Animals were randomly divided into four groups of eight animals each for 60 days as follows: normal diet, hypercholesterolemic diet (1% cholesterol), hypercholesterolemic diet (1% cholesterol) + sesame seed (10%), and hypercholesterolemic diet (1% cholesterol) + sesame oil (5%). Serum concentrations of total cholesterol, LDL-C, HDL-C, triglycerides, apoA and apoB, SGOT, SGPT, glucose and insulin were measured at the end of supplementation period in all studied groups. Hypercholesterolemic feeding resulted in a significant elevation of TC, TG, LDL-C, HDL-C, SGOT and SGPT as compared to the normocholesterolemic diet group (*P* < 0.05). Supplementation with sesame seed did not cause any significant alteration in lipid profile parameters, apolipoproteins, hepatic transaminases, glucose and insulin as compared to the hypercholesterolemic diet group (*P* > 0.05). In contrast, rabbits supplemented with sesame oil were found to have lower circulating concentrations of TC, LDL-C, HDL-C, SGOT and SGPT (*P* < 0.05), whilst concentrations of TG, apoA, apoB, insulin and glucose remained unaltered compared to the hypercholesterolemic diet group (*P* > 0.05). Supplementation with sesame oil, but not sesame seed, can ameliorate serum levels of lipids and hepatic enzymes in rabbits under a high-fat diet.

## 1. Introduction

Cardiovascular and cerebrovascular diseases are the most frequent causes of morbidity and mortality in the US and other western countries [1]. The primary cause of these vascular diseases is atherosclerosis and subsequent formation of lesions inside the coronary and cerebral arteries [2]. Atherogenic plaques are formed as a result of accumulation of lipids and fibrous elements in the subendothelial space of large arteries. Pathogenesis of atherosclerosis is multifactorial, and many modifiable and nonmodifiable risk factors have been identified [3]. These risk factors collectively contribute to the development, progression, and rupture of atherosclerotic plaque [4]. 

Recent decades have seen a rapid rise in reports indicating that botanical dietary supplements can improve cardiovascular health and prevent from atherosclerosis disease at several steps [5]. Current research into free radicals has confirmed that foods rich in antioxidants play an essential role in the prevention of cardiovascular disease and cancer [6–8]. Therefore, plant derived antioxidants are now receiving special attention [9, 10]. *Sesamum indicum* Linn. (sesame) belongs to the family Pedaliaceae and has been routinely used for culinary purposes in the oriental cuisine. In addition, several lines of evidence from traditional as well as modern medicine have confirmed various medicinal properties of sesame [11–13]. This plant possesses significant amounts of diverse phytochemicals, most importantly phenolic acids and lignans [14]. Oils and phytochemicals obtained from sesame have been shown to serve as promising natural antioxidants for both food preservation and medicinal applications [12]. 

Sesame seed contains moisture, crude oil, crude proteins, carbohydrates, crude fiber, and ash. Sesame oil (constituting ~50% of total seed content) is a rich source of mono- and polyunsaturated fatty acids [15]. Previous studies have reported that high antioxidant properties of sesame seed appear to be related to its main lignans namely sesamol, sesamolinol, pinoresinol, and sesaminol [16–18] as well as vitamin E [19]. As key constituents of sesame, lignans, dietary fibers and, polyunsaturated fatty acids have been reported to possess antihyperlipidemic and antihyperglycemic effects [20–22]. However, only a few studies have been conducted to clarify these pharmacological effects in hypercholesterolemic models supplemented with sesame powder and sesame oil [23–26]. Therefore, the present study was designed with the aim of evaluating the effects of sesame seed and sesame oil on serum lipids, apolipoproteins, liver enzymes, glucose, and insulin in a hyperlipidemic rabbit model. 

## 2. Materials and Methods

### 2.1. Sesame Seed Collection

Sesame seeds were purchased from a local fruit market in Shahrekord, Iran. Genus and species of the plant were identified and authenticated by a plant taxonomist and deposited at the Shahrekord University of Medical Sciences (Shahrekord, Iran, herbarium no. 409). Sesame oil was mechanically extracted using a screw press (oil expeller) as commonly used and described elsewhere [27].

### 2.2. Phytochemical Analysis

Total phenolic and total flavonoid contents of sesame seed and sesame oil were colorimetrically determined using Folin-Ciocalteu and aluminium chloride reagents, respectively [25, 28, 29]. Total values of phenolics and flavonoids were expressed in terms of gallic acid equivalent (GAE) and rutin equivalent (RE) (in mg/g), respectively.

### 2.3. Grouping and Treatments

Thirty- two adult male rabbits of New Zealand strain weighing 1.25–2.50 kg were purchased from the Razi Institute (Karaj, Iran). Rabbits were maintained in animal stainless steel mesh-bottomed cages at the Medicinal Plants Research Center, Shahrekord University of Medical Sciences (Shahrekord, Iran), for two weeks at 21–24°C and 12 h light:dark cycle. Animals were fed a standard basal diet for 2 weeks for adaptation. Following the initial two weeks, nourishment was done by standard grain food purchased from Pars Animal Feed Company (Tehran, Iran), containing 15% protein, 40–50% carbohydrates, 2% vegetable fat, and 15–25% fiber. Animals were randomly divided into four groups of eight animals each to be fed with normal diet, hypercholesterolemic diet (1% cholesterol), hypercholesterolemic diet (1% cholesterol) + sesame seed (10%), or hypercholesterolemic diet (1% cholesterol) + sesame oil (5%). 

The study protocol was approved by the Medical Ethics Committee of the Isfahan Cardiovascular Research Center.

### 2.4. Biochemical Measurements

Fasted blood samples were collected to determine serum concentrations of lipid parameters (comprising total cholesterol (TC), high-density lipoprotein cholesterol (HDL-C), low-density lipoprotein cholesterol (LDL-C), and triglycerides (TG)), liver enzymes (comprising serum glutamate oxaloacetate transaminase (SGOT) and serum glutamate pyruvate transaminase (SGPT)), insulin, glucose, and apolipoproteins A (apo A) and B (apo B). Serum insulin level was determined with an ELISA method using a commercial kit (Monobind Inc., CA, USA). Other evaluated biochemical factors were measured by routine enzymatic methods using commercial kits (Pars Azmoon, Tehran, Iran) on a Hitachi 902 autoanalyzer (Tokyo, Japan).

### 2.5. Statistical Analysis

Statistical analyses were conducted using SPSS software version 13.0 (SPSS Inc., Chicago, IL, USA). Between-group comparisons of biochemical factors were carried out using Kruskal-Wallis test. *Post-hoc* multiple comparisons were made using Dunn's test. A *P* value of <0.05 was considered as statistically significant.

## 3. Results 

Phytochemical investigations on sesame seed powder revealed total phenolics, flavonoids, and flavonols to be 30.1, 84.1, and 68.7 mg/g, respectively. As for the sesame oil, the values were 17.2, 52 and 47.8 mg/g, respectively.

Feeding rabbits with a hypercholesterolemic diet containing 1% cholesterol for 8 weeks resulted in a significant elevation of TC, TG, LDL-C, HDL-C, SGOT, and SGPT as compared to the normocholesterolemic diet group (*P* < 0.05) (Tables 1 and 2). In contrast, serum concentrations of HDL-C, glucose, insulin, apo A, and apo B remained statistically unchanged between hypercholesterolemic and normocholesterolemic diet groups (*P* > 0.05) (Tables 1 and 2). 

Supplementation with sesame seed (10%) did not cause any significant alteration in lipid profile parameters, apolipoproteins, hepatic transaminases, glucose, and insulin as compared to the hypercholesterolemic diet group (*P* > 0.05). In comparison with the normocholesterolemic diet group, serum levels of TC, LDL-C, SGOT, and HDL-C were elevated in the sesame seed containing diet group (*P* > 0.05). Rabbits supplemented with sesame oil (5%) were found to have lower circulating concentrations of TC, LDL-C, HDL-C, SGOT, and SGPT (*P* < 0.05), whilst concentrations of TG, apo A, apo B, insulin, and glucose remained unaltered compared to the hypercholesterolemic diet group (*P* > 0.05). Comparison of sesame oil versus normocholesterolemic diet groups revealed an elevation of triglycerides and reduction in apo A concentrations in the sesame oil fed group (*P* < 0.05). None of the evaluated biochemical parameters did significantly differ between sesame seed and sesame oil supplemented groups (*P* > 0.05).

## 4. Discussion

Findings of the present study suggested that dietary supplementation with sesame oil significantly reduces TC and LDL-C concentrations in rabbits under a lipogenic diet. These findings are consistent with those of previous studies. Visavadiya and Narasimhacharya [30] examined the effects of supplementation with sesame seed powder at 5% and 10% doses along with either normal or hypercholesterolemic diet for a period of 4 weeks. Administration of sesame seed powder to hypercholesterolemic rats resulted in a significant decline in plasma and hepatic total lipid and cholesterol, and plasma LDL-C whilst increasing HDL-C concentrations. In another investigation to evaluate hypocholesterolemic and antioxidant activity of sesame protein isolate, Biswas et al. [31] fed 18% sesame protein isolate with or without 2% cholesterol in comparison with casein to rats for 28 days. The results revealed that dietary sesame protein isolate reduces plasma total cholesterol, triacylglycerol, and LDL-C, increases HDL-C, and mitigates lipid peroxidation in both hypercholesterolemia and normocholesterolemic diet groups. Sirato-Yasumoto and associates demonstrated that supplementation with lignan-rich sesame has a remarkable potentiating effect on hepatic fatty acid oxidation while downregulating the activity of lipogenic enzymes. These favorable metabolic effects of lignan-rich sesame were reported to be accompanied with a profound hypotriglyceridemic effect [32]. In a recent report, Asgari et al. [25] investigated the protective effects of sesame on postprandial lipemic and glycemic response as well as circulating concentrations of endothelial function biomarkers in hypercholesterolemic rabbits. Their results revealed that sesame supplementation is associated with significant declines in serum TC, LDL-C, SGPT, and fibrinogen. In the study by Kumar et al. [33], the hepatoprotective activity of ethanolic extracts of sesame seeds (400 mg/kg and 700 mg/kg orally, once daily) were evaluated against carbon tetrachloride (CCL_4_)-induced hepatotoxicity in rats. The authors reported that substantially elevated serum enzymatic activities of SGOT and SGPT were significantly restored towards normalization by the extract. The results of this latter study strongly indicate that sesame seed extract has potent hepatoprotective effects against CCL_4_-induced hepatic damage in rats. These antihyperlipidemic effects of sesame oil could be attributed to the induction of cholesterol turnover via increasing fecal sterol excretion and hepatic bile acid production. Moreover, supplementation with sesame has been shown to improve antioxidant capacity through promoting hepatic catalase and superoxide dismutase activities [34]. 

Sesame contains a diversity of phytochemicals including lignans, polyphenolic and flavonoid compounds, dietary fibers, PUFA, and lecithin, which all possess documented antihyperlipidemic functions [21, 22, 30]. Different lignan derivatives of sesame such as sesamin, sesamol, sesaminol, sesaminolinol, and pinoresinol possess antioxidant functions and can prevent against membrane lipid peroxidation, ADP-Fe3+/NADPH-induced microsomal lipid peroxidation and Cu^2+^-induced LDL oxidation [35, 36] Furthermore, vitamin E and flavonoids that naturally occur in sesame have been reported to possess antioxidant and lipid-lowering properties [20, 31, 37, 38]. Sesamin is one of the most important lignan components of sesame seeds. This phytochemical has been reported to exert hypocholesterolemic effects through inhibition of the intestinal absorption of cholesterol, increase of biliary cholesterol excretion, and downregulation of 3-hydroxy-3-methylglutaryl coenzyme A reductase activity [39]. Aside from the aforementioned effects, sesamin has promising antioxidant properties which are, at least in part, due to the inhibition of tocopherol catabolism and enhancement of circulating as well as tissue concentrations of tocopherols [40]. The higher antihyperlipidemic activity of sesame oil versus sesame seed that was observed in the present study is most likely due to the higher frequencies of the aforementioned phytonutrients in the oil due to their lipophilic characteristics.

## 5. Conclusions

In summary, findings arising from the present study revealed a trend towards amelioration of lipid profile and hepatic enzymes following supplementation with sesame oil (5%). These positive effects of sesame oil further corroborate previous findings and jointly postulate therapeutic value of sesame oil as a safe and effective supplement for patients with dyslipidemia or nonalcoholic fatty liver disease. Future research should concentrate on the exact mechanisms underlying the antihyperlipidemic effects and also further explore whether these effects are exerted by a single constituent or are due to a synergism between different phytochemicals. 

## Figures and Tables

**Table 1:** Effects of *Sesamum indicum *on serum concentrations of lipid profile parameters and apolipoprotein in experimental groups.

Biochemical factor	Normal diet	Hypercholesterolemic diet (1% cholesterol)	Hypercholesterolemic diet (1% cholesterol) + sesame seed (10%)	Hypercholesterolemic diet (1% cholesterol) + sesame oil (5%)
Total cholesterol (mg/dL)	178 ± 19.889	2649 ± 610.58^a^	2045 ± 567.04^a^	1367 ± 503.00^b^
Triglyceride (mg/dL)	219 ± 54.488	1270 ± 651.77^a^	787 ± 280.30	1391 ± 386.00^a^
Low-density lipoprotein (mg/dL)	101 ± 10.909	1337 ± 546.05^a^	1279 ± 543.53^a^	570 ± 446.93^b^
High-density lipoprotein (mg/dL)	42 ± 2.754	1070 ± 292.45^a^	630 ± 59.711^a^	410 ± 95.394^b^
Apo lipoprotein A (mg/dL)	55 ± 3.697	39 ± 13.371	45 ± 12.703	36 ± 8.192^a^
Apo lipoprotein B (mg/dL)	24 ± 5.315	31 ± 10.068	29 ± 16.906	27 ± 6.595

^a^Significant difference with the normal diet group (*P* < 0.05).

^b^Significant difference with the hypercholesterolemic diet group (*P* < 0.05).

**Table 2:** Effects of *Sesamum indicum *on serum concentrations of liver enzymes, glucose, and insulin in experimental groups.

Biochemical factor	Normal diet	Hypercholesterolemic diet (1% cholesterol)	Hypercholesterolemic diet (1% cholesterol) + sesame seed (10%)	Hypercholesterolemic diet (1% cholesterol) + sesame oil (5%)
SGOT (U/L)	33 ± 3.096	105 ± 32.780^a^	92 ± 55.435^a^	58 ± 14.125^b^
SGPT (U/L)	40 ± 5.099	91 ± 18.924^a^	46 ± 28.849	56 ± 18.026^b^
Glucose (mg/dL)	132 ± 33.340	147 ± 74.637	151 ± 17.213	150 ± 40.329
Insulin (pmol/L)	26 ± 25.891	27 ± 5.935	27 ± 1.716	32 ± 830.39

SGOT: serum glutamate oxaloacetate transaminase; SGPT: serum glutamate pyruvate transaminase.

^a^Significant difference with the normal diet group (*P* < 0.05).

^b^Significant difference with the hypercholesterolemic diet group (*P* < 0.05).
